# Supplementary material for: Effective Coping with Academic Stress Is a Matter of Personality Types: Revisiting the Person-Centred Approach
Source: Behav Sci (Basel). 2023 Aug 18;13(8):687. doi: 10.3390/bs13080687 (PMC10451618; doi:10.3390/bs13080687)
Supplement: Supplementary file 1 [file behavsci-13-00687-s001.zip › behavsci-2533251-supplementary.pdf]

Supplementary Material

Table S1. Reliability Indexes of Internal Consistency, Cronbach's α, and McDonald's ω for the Coping Strategies Inventory (CSI) and the NEO-FFI.

Scale	Dimension	ω	95% CI	α	95% CI
NEO-FFI	Neuroticism	.90	[.89 - .91]	.89	[.88 - .90]
	Extraversion	.90	[.89 - .91]	.90	[.89 - .91]
	Openness	.79	[.77 - .81]	.79	[.76 - .81]
	Agreeableness	.74	[.71 - .77]	.73	[.71 - .76]
	Conscientiousness	.88	[.87 - .90]	.88	[.87 - .89]
CSI	Problem-Solving	.88	[.87 - .90]	.88	[.87 - .90]
	Self-Criticism	.91	[.90 - .92]	.91	[.90 - .92]
	Emotional Expression	.87	[.85 - .88]	.86	[.84 - .87]
	Wishful Thinking	.84	[.82 - .86]	.83	[.81 - .85]
	Social Support	.81	[.78 - .83]	.80	[.78 - .83]
	Cognitive Restructuring	.85	[.83 - .87]	.85	[.83 - .86]
	Problem Avoidance	.72	[.69 - .75]	.71	[.68 - .74]
	Social Withdrawal	.78	[.75 - .80]	.78	[.75 - .80]
